# The Role of Blood Inflammatory Markers in Salivary Gland Carcinoma: A Scoping Review

**DOI:** 10.3390/jcm14051762

**Published:** 2025-03-06

**Authors:** Andrea Migliorelli, Marianna Manuelli, Andrea Ciorba, Francesco Stomeo, Stefano Pelucchi, Chiara Bianchini

**Affiliations:** ENT & Audiology Unit, Department of Neurosciences, University Hospital of Ferrara, 44100 Ferrara, Italy

**Keywords:** inflammatory biomarker, malignant salivary gland tumors, NLR, PLR, salivary gland cancer, SII, SIRI

## Abstract

**Background/Objectives**: Malignant carcinomas of the salivary glands account for approximately 1 to 7% of all head and neck malignancies and approximately 0.3% of all malignant neoplasms. Recently, the scientific community has focused on finding biomarkers that could tailor the treatment for patients with this type of cancer. The neutrophil–lymphocyte ratio (NLR) was the first marker studied and it is one of the most widely used; the platelet–lymphocyte ratio (PLR), the systemic immune inflammation index (SII) and the systemic inflammatory response index (SIRI) have recently emerged as important biomarkers. The aim of this scoping review is to evaluate the role of inflammatory biomarkers in the management of salivary gland malignancies. **Methods**: A review of the English literature on inflammatory blood markers in major salivary gland cancer was performed using PubMed, Scopus, and Google Scholar databases. The literature review was performed using the Preferred Reporting Items for Systematic Reviews and Meta-analysis (PRISMA) guidelines for scoping reviews. **Results**: Eleven full-text articles were included in this review, for a total of 1356 patients in which the role of inflammatory biomarkers (NLR, PLR, SII or SIRI) for the diagnosis and prognosis of salivary gland cancer was assessed. NLR (i) was evaluated in all the studies; (ii) it contributed to the diagnosis and prognosis of both adult and pediatric patients and (iii) can be considered the main biomarker, even if a universal cut-off range is not available yet. PLR, SII and SIRI were introduced more recently and were evaluated only in some studies. **Conclusions**: The findings of this study suggest that elevated NLR values, regardless of age, are more frequently associated with malignancy and a poor prognosis. Further studies are necessary to evaluate the role of biomarkers other than NLR, and to identify universal and practical cut-off values.

## 1. Introduction

The incidence of malignant salivary gland tumors is 0.5–2 per 100,000, accounting for approximately 0.3% of all cancers, 1–7% of head and neck cancers, and 20% of all salivary gland neoplasms [1]. These tumors predominantly affect males between the ages of 40 and 60. The parotid gland is the most common site of salivary neoplasms; however, only 25% are malignant [2,3]. Nonetheless, the probability of finding a malignant tumor increases to 40, 50 and 90%, respectively, when considering the submandibular, minor salivary and sublingual glands. The survival rate of these tumors varies depending on the histological features, grading and tumor stage [2,3]. Furthermore, although rare, approximately 10–50% of pediatric parotid neoplasms have a malignant histology, with mucoepidermoid carcinoma representing the most common histotype [4,5,6]. Since this is a rare disease, there is still no consensus on the treatment approach. Moreover, the prognosis differs from that of the adult population and it is determined by tumor stage, histological grading and adverse pathological features [7]. In 2017, the World Health Organization officially recognized 24 different malignant histotypes of salivary gland tumors; the most common are mucoepidermoid carcinoma, acinic cell carcinoma, cystic adenoid carcinoma, pleomorphic ex-adenoma carcinoma and adenocarcinoma. In addition to the malignant histotypes, 11 benign histotypes were also recognized, including pleomorphic adenoma, Warthin’s tumor and basal cell adenoma [8].

The initial investigative procedures recommended for the evaluation of a salivary gland neoplasm include ultrasound and MRI, with cytological needle aspiration (FNAC) playing a pivotal role in assessing the nature of the lesion [9,10,11,12]. Most cancers have non-apparent malignant features; therefore, it is difficult to distinguish them, clinically, from benign tumors. Imaging techniques such as ultrasound and MRI can help to further define the tumor and are essential for staging; in particular, MRI can provide an accurate description and characterization of the lesion [9].

At the 39th European Congress of Cytology in Milan in 2015, the “Milan system” for reporting salivary gland cytopathology was established. This system provides a framework for the diagnosis and management of salivary gland neoplasms, with the cytological risk of malignancy being a fundamental consideration. The Milan system is categorized into six distinct categories: (i) non-diagnostic, (ii) non-neoplastic, (iii) atypia of undetermined significance, (iv) neoplasm, subdivided into (iva) benign neoplasm and (ivb) neoplasm with uncertain potential for malignancy, (v) suspected for malignancy, and (vi) malignant [13]. The Milan system has facilitated risk stratification in the cytopathology and management of these patients. Both cytology and imaging can provide useful support, but they are not always sufficient to guide an appropriate therapeutic decision and to provide reliable prognostic information [14]. Consequently, the possible identification of preoperative biomarkers that allow the differentiation of malignant from benign lesions, and the prediction of survival and disease-free time in cases of malignant salivary gland tumors, could be of great importance [15,16]. The tumor microenvironment plays a pivotal role in tumor progression, exhibiting a continuous interaction with the tumor itself. Inflammatory cells are undoubtedly part of this complex interaction [17]. Several studies have evaluated the prognostic role of these biomarkers in head and neck squamous cell carcinoma, breast carcinoma and colon–rectal carcinoma [18,19,20]. The neutrophil–lymphocyte ratio (NLR) was the first marker studied and it is one of the most widely used; the platelet–lymphocyte ratio (PLR), the systemic immune inflammation index (SII)—obtained from the neutrophil count x monocyte/lymphocyte count—and the systemic inflammatory response index (SIRI)—obtained from the neutrophil x platelet/lymphocyte formula—are emerging as important markers [21].

The aim of this scoping review is to evaluate the role of inflammatory biomarkers in the management of salivary gland malignancies.

## 2. Materials and Methods

**Figure 1:**
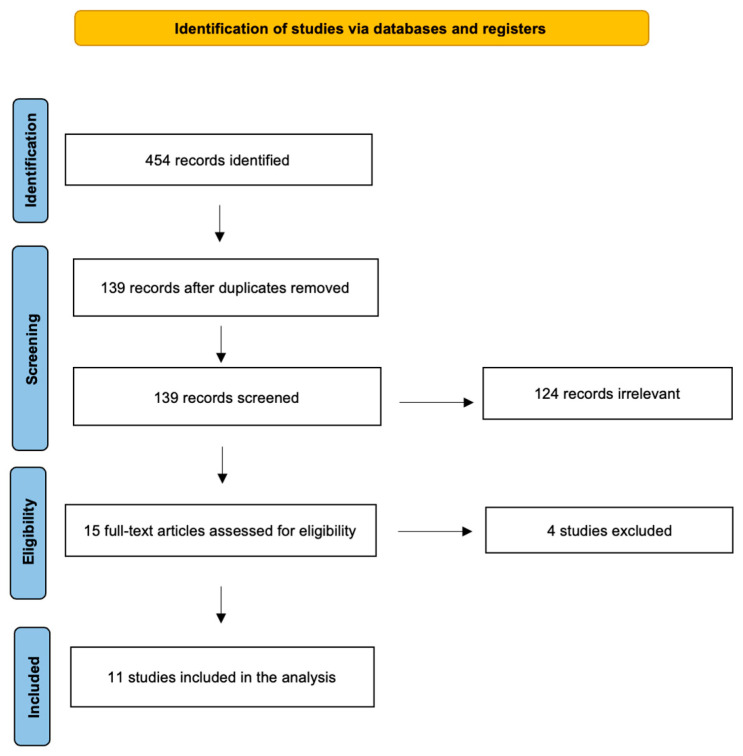
The literature review performed using PRISMA guidelines for scoping reviews.

A detailed review of the English-language literature on inflammatory blood markers in major salivary gland cancer was performed using PubMed/MEDLINE, EMBASE, and Cochrane Library databases. The search period was from January 2000 to December 2024. The terms used for the search were “salivary gland cancer” OR “salivary cancer” OR “parotid cancer” OR “submandibular gland cancer” OR “MSGTs” OR “salivary gland tumors” AND “systemic immune-inflammation index” OR “Inflammatory marker” OR “platelet-to-lymphocyte ratio” OR “neutrophil-to-lymphocyte ratio” OR “systemic inflammation response index” OR “PLR” OR “NLR” OR “SIRI” OR “SII”. The initial search yielded 454 candidate articles. It was performed according to the “Preferred Reporting Items for Systematic Reviews and Meta-Analyses” (PRISMA) guidelines for scoping reviews (Figure 1); as a scoping review, it cannot be registered on Prospero [22]. The inclusion criteria were as follows: (i) publication date after 2000; (ii) studies that aimed to use inflammatory blood markers in the management of salivary gland malignant tumors; and (iii) English language. Conference abstracts, case reports, and publications written in a language different from English were excluded. Papers only referring to benign salivary gland pathologies were also excluded.

Two authors (AM and MM) independently evaluated all titles, and relevant articles were individuated according to the inclusion/exclusion criteria; a senior author (CB) resolved any disagreements. At the end of the full-text review, 11 articles met the inclusion criteria [15,23,24,25,26,27,28,29,30,31,32].

## 3. Results

This scoping review includes 11 articles for a total of 1356 patients [15,23,24,25,26,27,28,29,30,31,32]. The main features of the analyzed studies and the major findings are summarized in Table 1 and Table 2, respectively. The articles originate from various countries, with a notable prevalence of China and Italy (four and three articles, respectively). The age range of the included patients varied from 6 to 87 years, with three studies focusing on the role of inflammatory markers in salivary gland carcinomas in the pediatric population [24,26,27].

Particularly considering the pediatric population, the most prevalent carcinoma observed in these patients is mucoepidermoid carcinoma of the parotid gland. A comprehensive analysis of existing studies revealed that the pretreatment NLR was predominantly evaluated as a prognostic marker. However, the statistical significance of its correlation with patient prognosis was not consistently demonstrated in all studies.

Considering the adult population, three studies evaluated the role of inflammatory factors in comparing malignant and benign salivary gland pathologies, while the others focused exclusively on malignant pathologies [15,30,32]. The main inflammatory marker used and considered in all the studies reviewed was the NLR, calculated as the ratio of neutrophils to lymphocytes. The cut-off value for this parameter varied considerably between 1.86 and 5. The second parameter used was the PLR (platelet-to-lymphocyte ratio), with a cut-off range of 129 to 187.6. Finally, only three studies utilized the SII (neutrophils multiplied by platelets and divided by lymphocytes) and SIRI (neutrophils multiplied by monocytes and divided by lymphocytes), with cut-off ranges of 594.91–917.585 and 0.94–2.045, respectively [29,30,32]. The discrepancy in these values can be attributed to the absence of a consensus methodology for calculating cut-off values. Some studies used the ROC curve and the Youden index, while others utilized the average value of the parameter. Demar et al. [15] showed that a high NLR can help to differentiate between low-grade and high-grade malignant tumors of the parotid gland. In addition, Cheng et al. [25] found that the NLR was significantly associated with tumor stage, disease stage, and disease grade.

In Table 3, the strengths and weaknesses of the analyzed studies are reported.

## 4. Discussion

This scoping review highlights the impact of the recent application of inflammatory biomarkers in the management of malignant salivary gland tumors.

The majority of the studies included were from China and Italy, with a highly variable mean age. The comprehensive analysis of 1356 cases revealed that the main inflammatory marker was the NLR, with cut-off values ranging from 1.86 to 5. Recent studies also included further biomarkers such as PLR (cut-off interval: 129 and 187.6), SII (594.91–917.585) and SIRI (0.94–2.045). The present study highlights the possible role of inflammatory biomarkers for (i) distinguishing between benign and malignant pathologies and (ii) as a prognostic indicator, particularly in the adult population. These markers could also have additional applications in pediatric parotid carcinomas [15,23,24,25,26,27,28,29,30,31,32].

In 1863, Rudolf Virchow was the first to propose an association between cancer and inflammation, highlighting the presence of inflammatory cells within tumors [33]. More recently, the importance of the systemic inflammatory response in promoting tumor cell proliferation, microvascular regeneration and distant metastasis has been further confirmed [34,35]. The immune microenvironment of a tumor is defined as the immune infiltrate that emerges alongside tumor growth, and several authors have confirmed its major role in tumor progression [36]. The systemic inflammatory response, promoted by the tumor itself and the tumor microenvironment, can lead to alterations in the circulating counts of lymphocytes, neutrophils, monocytes and platelets [37,38]. Neutrophils, in particular, have been reported to enhance a favorable microenvironment inducing local invasion and distant metastases [17]. Interleukin-6 (IL-6) and tumor necrosis factor alpha (TNF-α) induce neutrophilia by promoting the paraneoplastic production of myeloid growth factors by tumor cells [39,40]. Furthermore, tumor cells have been reported to secrete keratinocyte-derived chemokines, macrophage inflammatory protein 2 and Interleukin-8 (IL-8), which, in turn, can recruit neutrophils to the tumor site. The presence of these cells in the tumor microenvironment has been shown to contribute to the production of angiogenic factors, which can lead to lymphangiogenesis. In addition to the production of angiogenic factors, neutrophils within tumor microenvironments have been reported to produce vascular endothelial growth factor and IL-8 [41,42].

In contrast, lymphocytes have been identified as a key anti-tumor factor, playing a pivotal role in inducing tumor cells death and producing cytokines to mediate host immune responses, thereby inhibiting tumor proliferation [43]. As demonstrated by Fridman et al., the density of tumor-infiltrating CD8+ lymphocytes within the tumor microenvironment has been shown to be a favorable prognostic indicator in patients diagnosed with salivary gland cancer [44]. Regarding the proinflammatory environment, neutrophils have been observed to suppress the action of lymphocytes.

Consequently, the NLR has emerged as the most widely used biomarker, given that an increase in the NLR, determined by an increase in neutrophils and/or a decrease in lymphocytes, is generally associated with a poor prognosis [15,23,24,25,26,27,28,29,30,31,32]. This marker was utilized in all the studies analyzed in the present review.

Furthermore, tumor cells have been observed to protect themselves from lymphocyte attack by activating platelet aggregation. And platelets have been shown to play a significant role in tumor growth and distant metastatization [45,46]. Consequently, elevated PLR is also a negative prognostic factor.

In addition, novel inflammatory markers have been developed such as SII (neutrophils, lymphocytes and platelets) and SIRI (neutrophils, lymphocytes and monocytes) [29,30,32].

Damar et al. were the first to evaluate the role of the NLR in salivary gland tumors, finding that malignant tumors had a higher value than benign tumors. The authors attribute this discrepancy to an increase in neutrophils and a decrease in lymphocytes. A cut-off value of 1.86 was determined by the researchers [15]. In 2023, Committeri et al. found that both NLR and SIRI have greater accuracy in differentiating malignant from benign tumors [30]. Furthermore, in the same year, it was demonstrated that the combination of SIRI with the cytological result of FNAC increases preoperative diagnostic accuracy [32].

Consequently, a significant finding from our review is that inflammatory markers can play a pivotal role in enhancing the diagnostic accuracy of salivary gland tumors, particularly differentiating between malignant and benign pathologies. Nonetheless, only one study analyzed the relationship between inflammatory markers and cytology, finding that only SIRI can be positively integrated with cytological results [32]. Further multicenter studies are necessary to investigate the relationship between inflammatory parameters and cytological results.

Another significant application of these biomarkers is in the assessment of tumor prognosis. Cheng et al. found that a high NLR value before treatment is significantly associated with a worse prognosis. The authors demonstrated that the 10-year disease-specific survival rate was 68% in patients with an NLR < 2.48, decreasing to 58% in patients with a higher NLR [25]. However, Kawakita et al. observed that, in a group of patients with salivary ductal carcinoma, an elevated NLR (greater than 2.5) was associated with reduced overall survival, indicating an almost two-fold mortality risk [23]. Also, other parameters such as SII and SIRI were found to be statistically correlated with prognosis: specifically, the sum of SII and SIRI has been shown to independently predict patient survival after surgery [29].

In advanced malignant tumors, postoperative radiotherapy is recommended by guidelines. A significant complication associated with postoperative radiotherapy for parotid gland tumors is the occurrence of trismus [28]. A Turkish study performed in 2022 demonstrated that elevated preoperative NLR levels were associated with an increased incidence of trismus following adjuvant radiotherapy [28]. Moreover, in recurrent/metastatic tumors, a high preoperative NLR has been reported to be associated with a poor prognosis, both in terms of survival and recurrence, in patients treated with pembrolizumab [28,31]. These findings for malignant salivary gland tumors are consistent with those observed for head and neck cancers. In a study by Yu et al., it was reported that patients with head and neck cancer who had a high pretreatment NLR had a poor prognosis [47]. A further study of 285 head and neck cancer patients, treated by chemotherapy, found a relationship between a high NLR and advanced-stage hypopharyngeal or oropharyngeal carcinoma [48].

The preoperative prognostic significance of NLR was also evaluated in children diagnosed with parotid gland carcinoma. In this population, a high NLR value was found to have a poor prognosis in two of the three included studies [26,27]. Consequently, NLR may also serve as a marker in the case of pediatric salivary pathology. It is likely that, in the future, other biomarkers could also have a prognostic role in the pediatric population.

To date, a universally accepted cut-off level for NLR has not been identified yet, and this represents the main drawback to its application; while its significance in predicting malignancy and prognosis has been highlighted, its current clinical application is therefore still limited.

Even if these biomarkers are reliable, cost-effective and accessible tools that can be calculated through a simple blood sample, their main limitation is the absence of a universally accepted cut-off reference, as revealed by this review (see Table 1). A comparison between the various study findings is challenging, at present: to identify and standardize safe, reliable, and universally applicable cut-off values is crucial for the clinical application of these biomarkers in the near future.

## 5. Conclusions

To the best of our knowledge, this is the first review on the role of inflammatory biomarkers in the diagnosis and prognosis of salivary gland cancers. In particular, elevated NLR values, regardless of age, have been associated with malignancy and a poor prognosis. Other promising biomarkers, such as PLR, SII and SIRI, are also available; however, these have been employed in a limited number of studies.

In our opinion, further studies are necessary, particularly to investigate the role of biomarkers other than the NLR and to identify universal and functional cut-off values.

## Figures and Tables

**Table 1:** Identified papers on inflammatory blood markers and salivary gland cancer.

Authors (Yrs)	Country	NoP	Average Yrs (Range Yrs)	A/C	M/B	Parameters and Cut-Off	Cut-Off Calculation
Damar (2016) [15]	Turkey	182	53 (16–87)	A	M: 58 B: 124	NLR: 1.86	ROC
Kawakita (2017) [23]	Japan	140	64 (26–84)	A	M	NLR: 2.5 PLR: 186.2	ROC
Fang (2019) [24]	China	73	14.3 (8–18)	C	M	NLR: 2.48	Mean value
Cheng (2019) [25]	China	249	47.7 (19–73)	A	M	NLR: 2.48	Mean value
Seng (2019) [26]	China	123	14.3 (6–18)	C	M	NLR: 2.51	Mean value
Gao (2020) [27]	China	88	14.2 (6–18)	C	M	NLR: 2.32	Mean value
Somay (2022) [28]	Turkey	51	52 (31–75)	A	M	NLR: 2.7	ROC
Abbate (2022) [29]	Italy	74	56 (13–85)	A	M	NLR: 3.95 PLR: 187.6 SII: 917.585 SIRI: 2.045	ROC
Committeri (2023) [30]	Italy	117	Warthin: 62 Pleomorphic 52.5 Malignant 53.5	A	M: 28 B: 89	NLR: 3.62 PLR: 133.30 SII: 594.91 SIRI: 1.61	ROC
Lee (2023) [31]	USA	20	60 (41–68)	A	M	NLR: 5	Literature
Abbate (2023) [32]	Italy	239	55 (18–87)	A	M: 99 B: 140	NLR: 3.09 PLR: 129 SII: 788 SIRI: 0.94	ROC

Abbreviation legend: Yrs: years, NoP: numbers of patients, A/C: adults/children, M/B: malignant/benign, NLR: neutrophil–lymphocyte ratio, PLR: platelet–lymphocyte ratio, SII: systemic immune inflammation index, SIRI: systemic inflammatory response index.

**Table 2:** Identified papers: Objective and Major results.

Authors (Yrs)	Objective	Major Results
Damar (2016) [15]	To evaluate the pretreatment role of NLR, percentages and leucocyte counts in benign and malignant salivary gland tumors.	Malignant tumors showed higher NLR and neutrophil percentages, and lower lymphocyte percentages.
Kawakita (2017) [23]	To evaluate the role of NLR and PLR in OS and PFS in salivary duct carcinomas.	Multivariate analysis revealed a significant association between high NLR and OS; this association was not consistent with the PFS results. No notable associations were found between PLR and survival.
Fang (2019) [24]	To evaluate the long-term oncological outcome of pediatric patients with mucoepidermoid carcinoma of the parotid gland treated with total parotidectomy.	A high NLR was associated with recurrence in univariate analysis but not in the Cox model. High NLR was not linked to DSS in univariate analysis.
Cheng (2019) [25]	To assess the role of NLR in the prognosis of patients with parotid gland cancer.	NLR is an independent predictor of DSS, with high values indicating a worse prognosis.
Seng (2019) [26]	To assess the prognostic value of NLR in pediatric patients with parotid gland cancer.	In multivariate analysis, RFS and DSS rates were significantly reduced in patients with a high NLR.
Gao (2020) [27]	To evaluate the significance of pretreatment NLR in the prognosis of pediatric patients with parotid mucoepidermoid carcinoma.	Tumor histological grade and stage were significantly associated with NLR, and an NLR ≥ 2.32 was associated with a worse prognosis.
Somay (2022) [28]	To evaluate the significance of pretreatment NLR values for predicting radiation-induced trismus in parotid gland tumor patients treated with postoperative radiotherapy.	High pretreatment NLR levels are statistically predictive of increased rates of radiotherapy-induced trismus in patients with parotid carcinoma treated with surgery and adjuvant radiotherapy.
Abbate (2022) [29]	To investigate the predictive value of inflammatory biomarkers for OS in patients treated surgically for salivary gland malignancies.	SII plus SIRI can independently predict the OS of patients after surgery. The prognostic score based on these is useful in clinical decision making.
Committeri (2023) [30]	To increase the effectiveness of pre-surgical diagnosis and improve the differentiation between benign and malignant pathologies in salivary gland tumors using inflammatory biomarkers.	The inflammatory biomarkers NLR, PLR, SII and SIRI showed an accuracy of 0.88, 0.74, 0.76 and 0.83, respectively, in differentiating Warthin tumors from pleomorphic adenoma and malignant neoplasms.
Lee (2023) [31]	To investigate the association between pretreatment NLR and immune checkpoint inhibitor outcomes in patients with recurrent/metastatic salivary gland carcinoma treated with pembrolizumab.	Multivariate Cox analysis shows that pretreatment NLR remained independently associated with 6-month PFS and 2-year OS. In Kaplan–Meier analysis, patients with NLR ≥ 5 had significantly worse 6-month and 2-year PFS and OS after starting pembrolizumab therapy.
Abbate (2023) [32]	To evaluate the diagnostic efficacy of inflammatory biomarkers compared to FNAC alone in salivary gland tumors.	Combining SIRI with cytological analysis significantly increases the sensitivity to 82.8%, allowing it to be used routinely to increase the accuracy of preoperative diagnosis.

Abbreviation legend: NLR: neutrophil–lymphocyte ratio, PLR: platelet–lymphocyte ratio, SII: systemic immune inflammation index, SIRI: systemic inflammatory response index, FNAC: cytological needle aspiration, OS: overall survival, PFS: progression-free survival, DSS: disease-specific survival, RSF: recurrence-free survival.

**Table 3:** Pros and cons of the included papers.

Authors (Yrs)	Pros	Cons
Damar (2016) [15]	Large sample size. First study to analyze the role of inflammatory markers in salivary gland tumors. Cut-off selection method.	Retrospective study. Use of NLR only. Disparity between malignant and benign tumors, with a reduced malignant sample.
Kawakita (2017) [23]	Large sample size. Multi-parameter analysis. Cut-off selection method.	Retrospective study.
Fang (2019) [24]	Large sample size.	Retrospective study. Use of NLR only. Cut-off selection method.
Cheng (2019) [25]	Large sample size.	Retrospective study. Use of NLR only. Cut-off selection method.
Seng (2019) [26]	Large sample size.	Retrospective study. Use of NLR only. Cut-off selection method.
Gao (2020) [27]	Large sample size.	Retrospective study. Use of NLR only. Cut-off selection method.
Somay (2022) [28]	Analyzing a specific subcategory. Cut-off selection method.	Retrospective study. Use of NLR only.
Abbate (2022) [29]	Multi-parameter analysis. Cut-off selection method.	Retrospective study.
Committeri (2023) [30]	Multi-parameter analysis. Cut-off selection method.	Retrospective study. Disparity between malignant and benign tumors, with a reduced malignant sample.
Lee (2023) [31]	Analyzing a specific subcategory.	Retrospective study. Use of NLR only. Cut-off selection method.
Abbate (2023) [32]	Multi-parameter analysis. Cut-off selection method. Large sample size.	Retrospective study.

Abbreviation legend: NLR: neutrophil–lymphocyte ratio.

## Data Availability

No new data were created in this study since it is a review paper; therefore, data sharing is not applicable.
